# A Single Peroxisomal Targeting Signal Mediates Matrix Protein Import in Diatoms

**DOI:** 10.1371/journal.pone.0025316

**Published:** 2011-09-22

**Authors:** Nicola H. Gonzalez, Gregor Felsner, Frederic D. Schramm, Andreas Klingl, Uwe-G. Maier, Kathrin Bolte

**Affiliations:** 1 Laboratory for Cell Biology, Philipps University of Marburg, Marburg, Germany; 2 LOEWE-Zentrum für Synthetische Mikrobiologie (SynMikro), Marburg, Germany; University of Melbourne, Australia

## Abstract

Peroxisomes are single membrane bound compartments. They are thought to be present in almost all eukaryotic cells, although the bulk of our knowledge about peroxisomes has been generated from only a handful of model organisms. Peroxisomal matrix proteins are synthesized cytosolically and posttranslationally imported into the peroxisomal matrix. The import is generally thought to be mediated by two different targeting signals. These are respectively recognized by the two import receptor proteins Pex5 and Pex7, which facilitate transport across the peroxisomal membrane. Here, we show the first *in vivo* localization studies of peroxisomes in a representative organism of the ecologically relevant group of diatoms using fluorescence and transmission electron microscopy. By expression of various homologous and heterologous fusion proteins we demonstrate that targeting of *Phaeodactylum tricornutum* peroxisomal matrix proteins is mediated only by PTS1 targeting signals, also for proteins that are in other systems imported via a PTS2 mode of action. Additional *in silico* analyses suggest this surprising finding may also apply to further diatoms. Our data suggest that loss of the PTS2 peroxisomal import signal is not reserved to *Caenorhabditis elegans* as a single exception, but has also occurred in evolutionary divergent organisms. Obviously, targeting switching from PTS2 to PTS1 across different major eukaryotic groups might have occurred for different reasons. Thus, our findings question the widespread assumption that import of peroxisomal matrix proteins is generally mediated by two different targeting signals. Our results implicate that there apparently must have been an event causing the loss of one targeting signal even in the group of diatoms. Different possibilities are discussed that indicate multiple reasons for the detected targeting switching from PTS2 to PTS1.

## Introduction

Peroxisomes constitute a ubiquitous family of cellular compartments and are widely distributed across the eukaryotic kingdom. Considered as compartment with special functions, they produce and/or detoxify many dangerous and harmful compounds within the peroxisomal matrix. Furthermore, they have been shown to fulfill a variety of biochemical and metabolic functions [1], which can differ substantially from species to species. As peroxisomes possess neither an intrinsic genome nor transcription and translation machineries, all matrix proteins have to be imported across the peroxisomal membrane after their synthesis in the cytosol. The import process is facilitated by the so called peroxisomal importomer [2], consisting of a variable number of so called peroxins (Pex) depending on the respective organism. Targeting and import of cytosolically expressed matrix proteins into peroxisomes generally depends on two different targeting signals, known as peroxisomal targeting signal (PTS) type 1 and type 2, respectively. The majority of peroxisomal matrix proteins are equipped with PTS1, which is located at the extreme C-terminus of the proteins [3], [4], [5]. Today it is known from PTS1 sequences of different examined proteins and organisms that they fit the consensus sequence (S/A/C)-(K/R/H)-(L/M) [6]. This sequence is recognized and bound by the Pex5 receptor protein [7], [8], [9], followed by subsequent targeting. Matrix proteins that are targeted into peroxisomes due to the presence of PTS2 sequences are much less frequent than those proteins targeted by PTS1s. PTS2 sequences are in contrast to PTS1s located N-terminally in peroxisomal matrix proteins [10]. Sequence comparisons led to the PTS2 consensus sequence (R/K)-(L/V/I)-X5-(H/Q)-(L/A) [11]. This sequence is recognized by the soluble receptor protein Pex7 [12], [13], [14].

Both, PTS1 and PTS2 targeted matrix proteins are recognized and bound by their respective receptor protein in the cytosol. After assembly of the receptor-cargo complex, it associates with the docking complex residing within the peroxisomal membrane, which consists of Pex13, Pex14 and – in *Saccharomyces cerevisiae* – also Pex17 [15]. Cargo translocation across the membrane is thought to be managed by a transient Pex5-dependent pore, when Pex5 receptor proteins change their conformational status from a cytosolic to a membrane-integrated form [16], [17]. Thus, all loaded cargo proteins traverse the peroxisomal membrane and are released into the matrix; a step which has yet to be characterized [6]. After disassembly, receptor proteins must be removed from the peroxisomal membrane into the cytosol. Here, ubiquitination via Pex4 and Ubc4 as well as the ubiquitin ligases Pex2, Pex10, Pex12 flags Pex5 proteins for membrane extraction by Pex1 and Pex6. This process is mediated in a mechanistic similar way to two other eukaryotic protein translocation systems, the endoplasmatic reticulum associated degradation (ERAD) machinery [18], [19] and SELMA (symbiont-specific ERAD-like machinery), a plastidal pre-protein translocation machinery [20].

The knowledge about peroxisomes including import of peroxisomal proteins, metabolic pathways, generation, division and maintenance is mostly examined in only a handful of species including mammals, yeasts and the model plant *Arabidopsis thaliana*. These organisms represent only two of the five major eukaryotic groups [21], the Unikonta and Plantae. There is still little information regarding the distribution, diversity and function of peroxisomes across the remaining major groups of eukaryotic organisms, the Excavata, Cercozoa/Rhizaria and Chromalveolata [22]. Chromalveolates join several of the major protist groups with much of the diversity of mostly photosynthetically active algae like dinoflagellates and diatoms. A characteristic feature of all chromalveolates is the existence of so called complex plastids [23], [24], [25], which might have been lost in the cases of the chromalveolate groups of ciliates and oomycetes [26].

Research data on basic features of peroxisomes in chromalveolates, including metabolism and protein import, is still extremely limited. There is histochemical and biochemical evidence for the presence of this compartment in the oomycete genus *Phytophthora* [27] and in the ciliates *Tetrahymena* and *Paramecium* [28], [29]. The apicomplexans, including the human pathogens *Plasmodium falciparum* and *Toxoplasma gondii*, are considered the first and only group devoid of peroxisomes in the presence of mitochondria [30]. The occurrence of peroxisomes in the ecologically relevant group of diatoms has been predicted from *in silico* data and detected by classic peroxisomal enzyme activity in enzymatic assays [31], [32].

Here we present the first *in vivo* data of peroxisomal distribution patterns in the model diatom *Phaeodactylum tricornutum* using fluorescence and electron microscopy. Our results indicate that this organism interestingly uses only PTS1 as single targeting signal to target matrix proteins into the compartments and thus could very well have lost the PTS2 import pathway.

## Results and Discussion

### 1. Peroxins and peroxisomal proteins of the diatom *P. tricornutum*

Screening the genome of the diatom *P. tricornutum* (http://genome.jgi-psf.org/Phatr2/Phatr2.home.html) revealed the existence of genes encoding predicted orthologs of peroxisomal proteins.

Beside peroxins necessary for peroxisomal biogenesis and enzymes of various peroxisomal pathways (table 1, 2), the *in silico* analyses uncovered the existence of components of a matrix protein import machinery. Most important in this regard was the identification of the PTS1 receptor protein Pex5 (PtPex5), which is essential for matrix protein import and corresponds to the identification of putative PTS1 signals in several peroxisomal enzymes (see later). Using the SMART prediction tool [33] PtPex5 is supposed to contain five tetratricopeptide (TPR) repeats at its C-terminus for binding different PTS1 cargo proteins.

**Table 1:** Peroxisomal proteins in *P. tricornutum*.

Peroxisomal Protein	Metabolic Pathway	PTS	Protein ID
catalase	detoxification	SKL	22418
bifunctional enzyme	beta-oxidation of fatty acids	SKL	55069
long chain acyl-CoA ligase	beta-oxidation of unsaturated fatty acids	SKL	17720
carnitine-o-acetyltransferase	beta-oxidation of fatty acids	SKL	48078
glycolate oxidase	glyoxylate cycle	SRL	22568
acyl-CoA oxidase	beta-oxidation of fatty acids	SRL^a^	19979
acyl-CoA dehydrogenase	beta-oxidation of fatty acids	SRL	42907
trans-2-enoyl-CoA reductase	fatty acid synthesis	ARL	37372
malate synthase	glyoxylate cycle	AKL	54478
3-keto acyl-CoA thiolase	beta-oxidation of fatty acids	SSL	41969

a putative targeting signal.

So far identified putative peroxisomal proteins of the diatom *P. tricornutum*, their metabolic affiliations and targeting signals.

**Table 2:** Peroxins in *P. tricornutum*.

Peroxin	Function	Protein ID
Pex1	AAA ATPase	14397
Pex2	peroxisomal ubiquitin ligase	49301
Pex3	localization and stabilization of peroxisomal membrane proteins	50623
Pex4	peroxisomal ubiquitin conjugating enzyme	47555
Pex5	signal receptor for PTS1 of peroxisomal matrix proteins	32173
Pex6	AAA ATPase	46568
Pex10	peroxisomal ubiquitin ligase	47516
Pex11	peroxisome division and proliferation	44128
Pex12	peroxisomal ubiquitin ligase	49405
Pex19	import receptor for newly-synthesized class I PMPs	31927

So far identified putative peroxins of the diatom *P. tricornutum* and their functions.

Interestingly, the typical docking complex needed for an import of matrix proteins, consisting of Pex13 and Pex14 [15], could not be identified. The lack of a gene coding for Pex13 in the diatom is not surprising, as it has been shown to be absent in the photosynthetic lineage [30] and has probably been replaced by another as yet unidentified membrane protein. The same might be true concerning the absence of Pex14 in *P. tricornutum*. According to their affiliation to the docking complex, Pex14 proteins have been shown to form the transient pore complex together with Pex5 in the peroxisomal membrane [17]. Thus, it will be interesting to investigate the composition of the docking complex and the putative transient pore complex in *P. tricornutum*, which might shed light on further heretofore unidentified components of the peroxisomal importomer in diatoms and perhaps in additional members of photosynthetic lineage.

The remaining peroxins identified have functions involved in ubiquitination, which is required for receptor release from the peroxisomal membrane [34]. Other than the ubiquitin-conjugating enzyme Pex4 and the three ubiquitin ligases Pex2, Pex10 and Pex12, the cytosolic ATPases Pex1 and Pex6 were attained with the afore mentioned *in silico* analyses (table 2), the latter of which are associated to the peroxisomal membrane by Pex15 in yeast [35] and by Pex26 in mammals [36]. Anchoring cytosolic AAA-ATPases to the membrane is probably accomplished by a variety of proteins in diatoms, as orthologs for neither Pex15 nor Pex26 were identified. This will require further clarification with analyses of the composition of the peroxisomal membrane proteome.

Concerning peroxisomal biogenesis and division, a Pex11 ortholog could be identified, which is thought to play a role in peroxisomal division [37].

Pex3 and Pex19 are known to facilitate transport and membrane integration of cytosolically translated peroxisomal membrane proteins (PMP) and are encoded on the *P. tricornutum* genome. Pex19 is the putative cytosolic PMP receptor protein [38] that recognizes and targets cargo proteins to the peroxisomal membrane, where they are inserted into the peroxisomal membrane in association with the intrinsic membrane protein Pex3 [39].

### 2. Peroxisomal matrix proteins lack PTS2-like sequences

During the course of *in silico* analyses, several enzymes were identified, mainly having an involvement in beta-oxidation of fatty acids but also having functions in the glyoxylate cyle (table 1). Catalase, the typical peroxisomal marker protein, is encoded as a single ortholog on the genome and is equipped with the typical PTS1 tripeptide SKL. Interestingly, the entirety of identified enzymes contains conserved tripartite targeting motifs or derivatives thereof at the C-terminal extremity of the proteins (table 1), whereas no PTS2-containing enzymes could be identified. The PTS2 receptor protein Pex7 seems also to be absent in *P. tricornutum*, and no putative orthologs could be identified, indicating that targeting of peroxisomal enzymes might be exclusively mediated by PTS1 or that import of putative but still not identified PTS2 harboring peroxisomal proteins is facilitated by another yet unknown receptor protein.

### 3. *In vivo* and *in situ* localization of putative peroxisomal proteins

**Figure 1:**
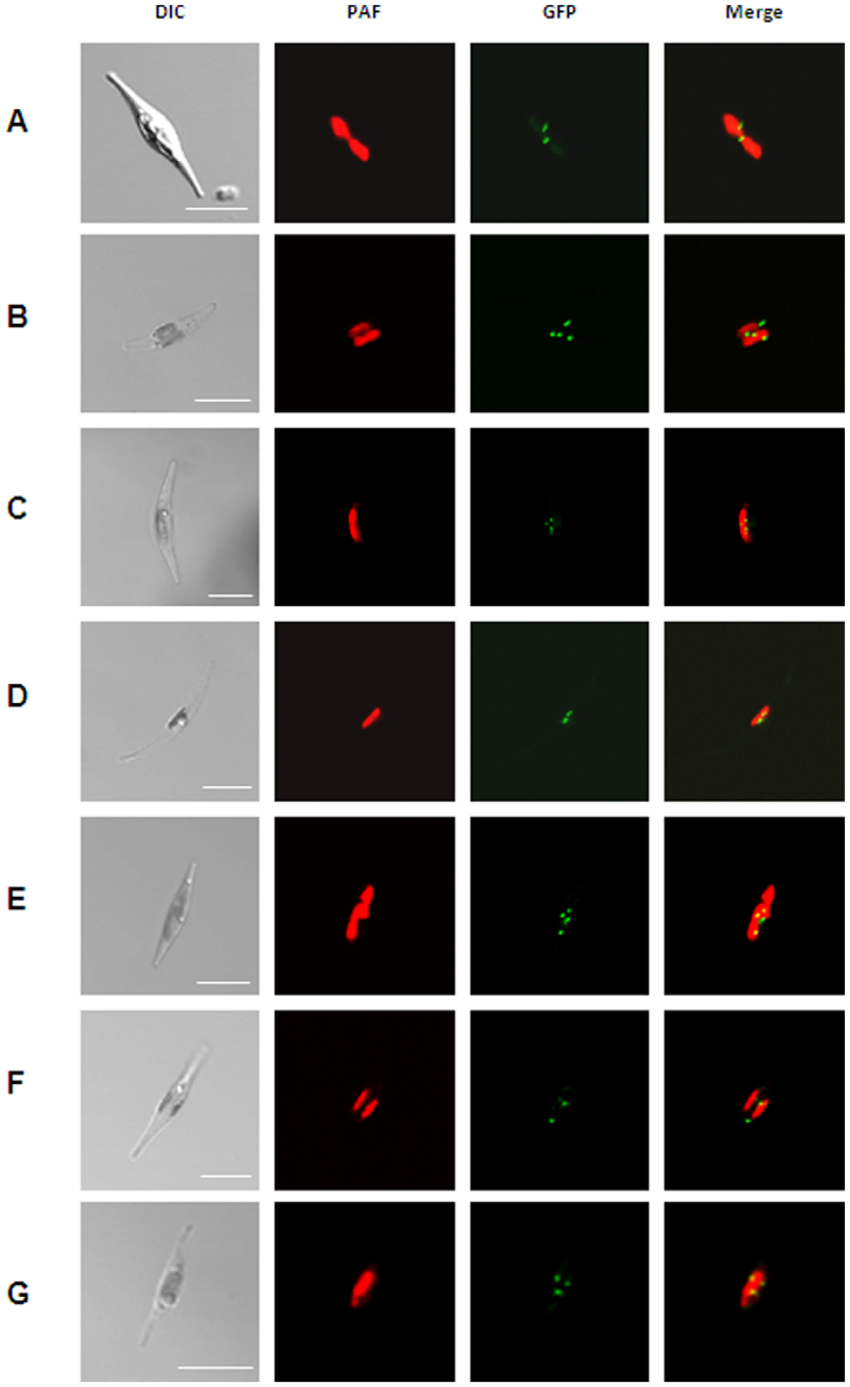
Localization studies of different putative peroxisomal proteins in *P. tricornutum*. (A) GFP-catalase, (B) GFP-long chain acyl-CoA ligase, (C) GFP-trans-2-enoyl-CoA reductase, (D) GFP-malate synthase, (E) GFP-3-ketoacyl-CoA thiolase, (F) Pex10-GFP and Pex3-GFP (G). All fusion proteins localize in punctate structures as indicated by the GFP fluorescence (green) next to the complex plastid (red), which is visualized due to the chlorophyll autofluorescence (red). PAF, plastid autofluorescence; GFP, GFP fluorescence. Scalebars represent 10 µm.

As, at the beginning of this study, no data regarding peroxisomal distribution patterns, number or size in diatoms, were available we initially investigated the localization patterns of several peroxisomal proteins. Therefore, enzymes with representative PTS1 variants, the integral membrane proteins Pex10 and Pex3 were expressed as GFP fusion proteins in *P. tricornutum* cells. PTS1 proteins were equipped N-terminally with GFP in which the targeting signal remained accessible, whereas Pex10 and Pex3 were fused C-terminally to GFP. All transfected cells – regardless of the expressed protein – showed similar punctate patterns of the detected GFP fluorescence (Fig. 1A–G). The number of putative, observed peroxisomes varies only slightly from three to five between microscopically examined clones.

**Figure 2:**
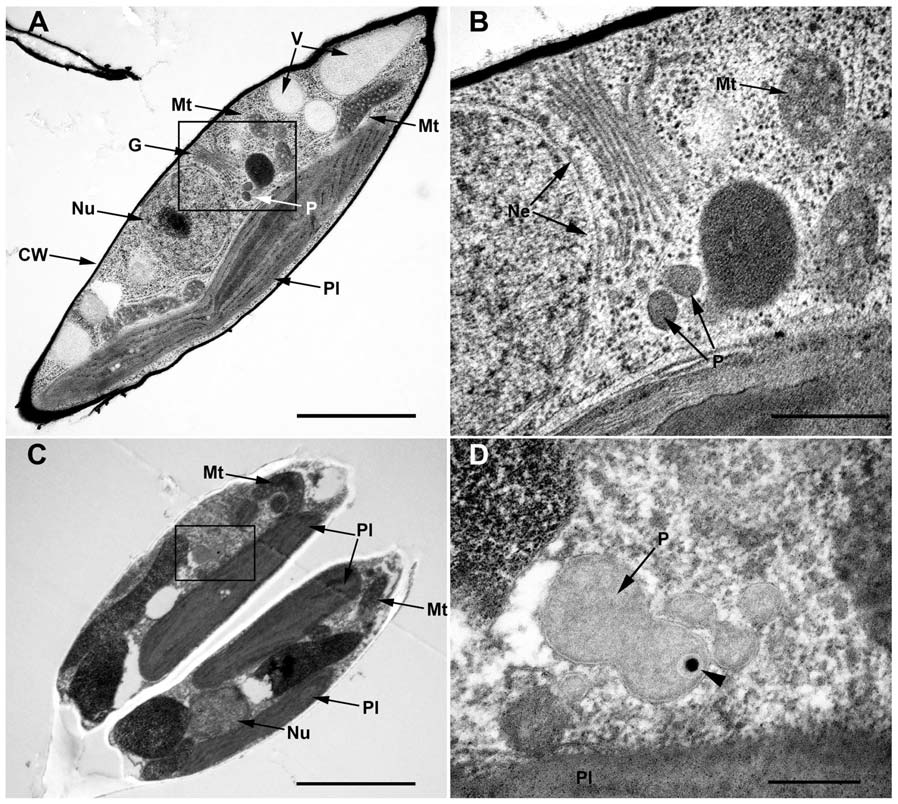
Immunolocalization studies of a peroxisomal GFP-fusion protein in *P. tricornutum*. (A) Ultrathin section of *P. tricornutum* expressing Pex10-GFP in Epon without antibody labeling. The boxed area is shown in (B) at higher magnification and illustrates two peroxisomes in proximity to the nucleus, the golgi and the plastid. (C) Immuno labeling of Pex10-GFP in a dividing *P. tricornutum* cell. (D) higher magnification. The 20 nm gold particle, coupled to the secondary antibody is visible within the peroxisome (arrow head), which is surrounded by a lipid bilayer. CW, cell wall; G, golgi apparatus; Mt, mitochondrium; Ne, nuclear envelope; Nu, nucleus; P, peroxisome; Pl, plastid; V, vacuole; arrow head, 20 nm gold. Scalebars represent 2 µm (A, C), 500 nm (B) and 200 nm (D).

To ensure that the punctuate structures observed in course of fluorescence microscopy are neither a result of mistargeting nor cytosolic protein accumulations, we performed electron microscopic studies on strains expressing different GFP fusion proteins. In general, peroxisomes appear as electron dense, membrane-bound compartments (Fig. 2A, B, S1). These structures could be confirmed as being peroxisomes due to immunolocalization studies as labeling of different peroxisomal GFP-fusion proteins with gold particles could be observed in these membrane-bound compartments (Fig. 2C, D, S2).

The identity of those compartments is further supported by a close association with the complex plastid as it has been already known from peroxisomes in *A. thaliana* [40]. This would be an advantageous localization of the peroxisomes due to an exchange of metabolites between peroxisomes and the complex plastid in course of photorespiration; a metabolic pathway mainly taking place in the chloroplast, the peroxisome and the mitochondrion [41]. Most of photorespiratory enzymes in *P. tricornutum* have been already identified during an identification and annotation process of genes involved in carbon acquisition and metabolism [42]. These include peroxisomal homologs, like a glycolate oxidase, a serin-glyoxylat transaminase and a glutamate-glyoxylat aminotransferase, which convert in several enzymatic steps glycolat to glycine. Due to some unexpected predictions of protein localization, it was concluded that the photorespiration pathway in diatoms possesses some differences in comparison to land plants [42], which is an interesting topic for future research.

### 4. Matrix protein import is mediated by only one single targeting signal

Surprisingly, the genome of *P. tricornutum* seems to lack all genes encoding proteins specific for the PTS2 import pathway, the most important of which is the PTS2 receptor protein Pex7. For full activity, Pex7 proteins require additional soluble proteins, which are not well conserved among eukaryotic organisms. These so-called PTS2 co-receptors, including Pex18, Pex20 and Pex21, are species-specific proteins [43], [44]. In mammalians two different isoforms of the PTS1 receptor Pex5 function as co-receptors of Pex7 in the PTS2 import pathway, and in the case of *A. thaliana* the distinct receptor itself even acts as a co-receptor [45], [46]. It is obvious that these different PTS2 co-receptors are highly divergent because of their low sequence similarities. We could not identify either homologous proteins of Pex18, Pex20 or Pex21, nor do EST data support the premise that different splice variants of PtPex5 exist.

An absence of the import receptor Pex7 is supported by the fact that orthologous peroxisomal proteins, which in other organisms typically contain a PTS2, can be described by one of the following scenarios. These proteins are 1) equipped in *P. tricornutum* with a C-terminal PTS1, 2) predicted mitochondrial derivates, or 3) assumed to be cytosolically located due to the lack of any identifiable targeting sequence (table 3). Scenario 1 applies to the beta-oxidation enzymes 3-ketoacyl-CoA thiolase and acyl-CoA oxidase, as typical PTS1s were identified in these proteins instead of PTS2s. Other proteins, e.g. orthologs of a citrate synthase are known to be targeted to peroxisomes via PTS2 in several organisms but are predicted to have a cytosolic and mitochondrial localization in *P. tricornutum* [42].

**Table 3:** Peroxisomal proteins from different organisms representing the major eukaryotic groups and their predicted targeting signals.

eukaroytic group	organism	Pex7	3-ketoacyl-CoA thiolase	acyl-CoA oxidase	citrate synthase	malate dehydrogenase
Chromalveolata	*Phaeodactylum tricornutum*	—	PTS1	PTS1	x	x
	*Thalassiosira pseudonana*	—	PTS1	PTS1	x	PTS1
	*Fragilariopsis cylindrus*	—	PTS1	PTS1	x	x
	*Ectocarpus siliculosus*	yes	PTS2	x	x	x
	*Phytophthora infestans T30-4*	yes	PTS2	PTS2	x	PTS1
	*Perkinsus marinus*	yes	PTS2	PTS1	x	x
	*Tetrahymena thermophila*	yes	PTS2	PTS2	PTS1, PTS2	PTS2
Plantae	*Cyanidioschyzon merolae*	—	PTS1^a^	PTS1	PTS1	x
	*Chlamydomonas reinhardtii*	yes	PTS2	PTS1	PTS2	PTS2
	*Volvox carteri*	yes	PTS2	PTS2	PTS2	PTS2
	*Physcomitrella patens*	yes	PTS2	PTS1	PTS1, PTS2	PTS2
	*Arabidopsis thaliana*	yes	PTS2	PTS1, PTS2	PTS1, PTS2	PTS2
Excavata	*Naegleria gruberi*	yes	PTS1	PTS2	x	x
Unikonta	*Trichoplax adhaerens*	yes	PTS2	PTS1	x	x
	*Dictyostelium discoideum*	yes	PTS2	PTS1, PTS2	PTS2	PTS2
	*Saccharomyces cerevisiae*	yes	PTS2	b	PTS1	PTS1
	*Ustilago maydis*	yes	PTS2	PTS1	x	x
	*Caenorhabditis elegans*	—	PTS1	PTS1	PTS1	x
	*Danio rerio*	yes	PTS2	PTS1	x	x
	*Mus musculus*	yes	PTS2	PTS1	x	x
	*Rattus norvegicus*	yes	PTS2	PTS1	x	x

a possible unkown derivat AAL of the conventional PTS1 consensus sequence.

b the peroxisomal acyl-CoA oxidase of *S. cerevisiae* lacks both PTS1 and PTS2 targeting signal [60].

It should be noted that currently no genome data of a rhizarian organism is available. PTS1, peroxisomal targeting signal 1; PTS2, peroxisomal targeting signal 2; x, no peroxisomal orthologs identified; —, no orthologs identified.

**Figure 3:**
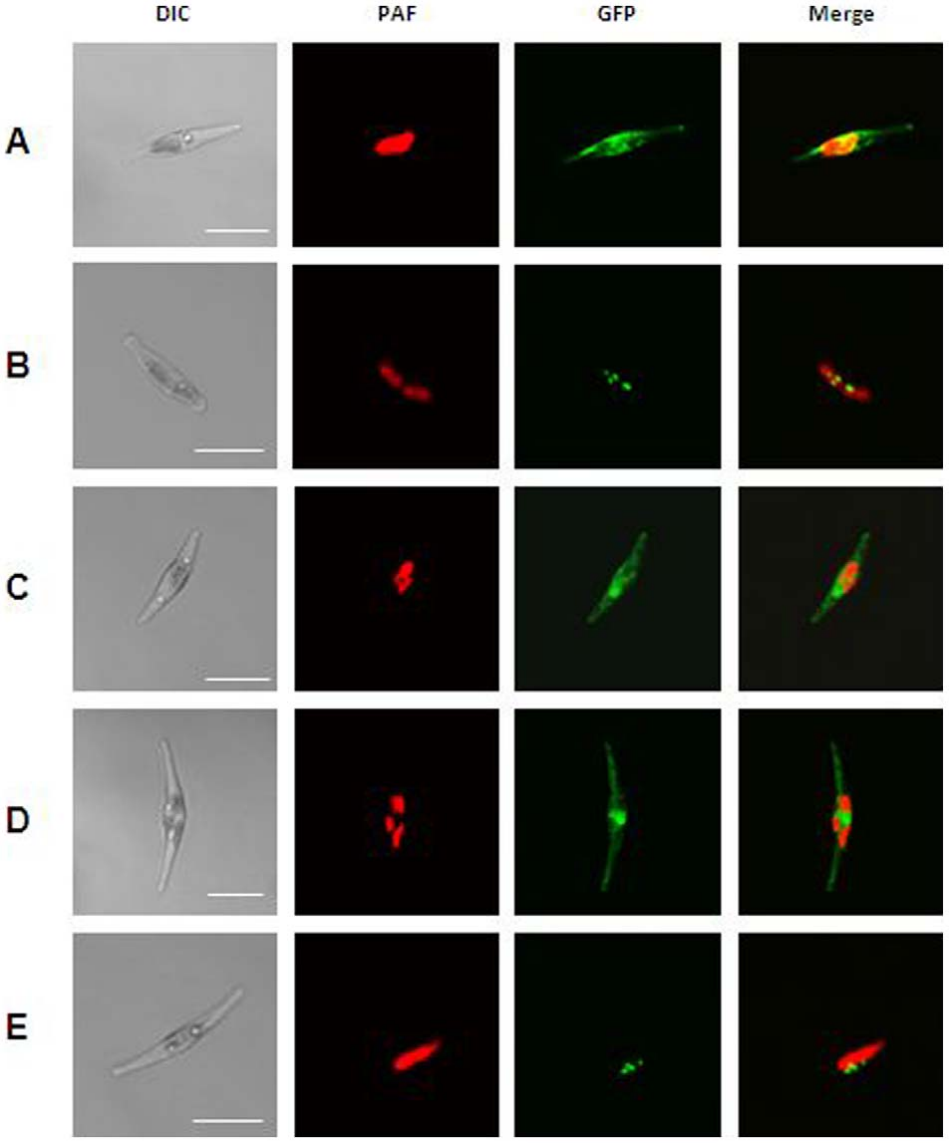
Localization studies confirming the absence of the PTS2 import pathway in *P. tricornutum*. Localization of *A.t* 3-ketoacyl-CoA thiolase wt (A) and equipped with the PTS1 SKL (B) in *P. tricornutum*; (C) *Pt* 3-ketoacyl-CoA thiolase upon deletion of its C-terminal tripeptide SSL; (D) GFP equipped with PTS2 (RLQVVLGHL) N-terminal and (E) PTS1 (SKL) C-terminal. PAF, plastid autofluorescence; GFP, GFP fluorescence. Scalebars represent 10 µm.

To test, whether the entire targeting pathway of PTS2 is indeed absent in *P. tricornutum*, we expressed the orthologous protein 3-ketoacyl-CoA thiolase from the model plant *A. thaliana* (accession no. AEC08791) fused C-terminally with GFP, heterologously in the diatom. This orthologous protein has previously been shown to be targeted to peroxisomes in *A. thaliana* suspension cells due to its PTS2 [47]. Remarkably, all clones resulted in clear cytosolic GFP fluorescence arising from the fusion proteins, and no punctate fluorescence indicative of putative peroxisomal structures was observed (Fig. 3A). This is indicative of *P. tricornutum*'s inability to import PTS2-targeted proteins into its peroxisomes. To exclude mistargeting during heterologous expression, the protein was equipped with the typical C-terminal PTS1 tripeptide SKL and was N-terminally fused to GFP. Such fusion proteins were observed in punctate putative peroxisomal structures in *P. tricornutum* cells (Fig. 3B).

The thiolase of *P. tricornutum* possesses a variant of the classical C-terminal PTS1 consensus sequence and was shown to be targeted to the putative peroxisomal structures (Fig. 1D). Upon deletion of the C-terminal tripeptide SSL, a cytosolic localization was observed (Fig. 3C). Thus, it can be concluded that the PTS1 variant of 3-ketoacyl-CoA thiolase of *P. tricornutum* is responsible for targeting the protein to peroxisomal structures. The tripeptide SSL has been already shown to function as a C-terminal peroxisomal targeting signal in *A. thaliana* [48]. Furthermore, we fused a typical PTS2 consensus sequence (RLQVVLGHL) N-terminally to GFP. This PTS2-equipped GFP has been previously shown to be imported into peroxisomes in human fibroblasts as well as in *S. cerevisiae* as in those organisms the classical import of PTS2 proteins is present [49]. However, transfected diatom cells clearly showed a cytosolic GFP localization of this expressed PTS2 proteins (Fig. 3D), confirming the diatom's inability to target PTS2 proteins into peroxisomal structures once again. As a control, GFP was C-terminally equipped with the tripeptide SKL and was then shown to be localized in peroxisomal structures (Fig. 3E). Taken together, these experiments confirm the deficiency of the receptor protein Pex7, which is known in other organisms to be necessary for conserved PTS2 import pathway.

### The PTS2 import pathway seems to be absent not only in *P. tricornutum*

The occurrence of PTS2 signals in peroxisomal matrix proteins known to be generally restricted to only a few enzymes in many organisms [10]. Representative organisms of all major eukaryotic groups have been shown to possess at least one typical PTS2 harboring peroxisomal protein (table 3). The only known exception to this trend is the nematode *Caenorhabditis elegans*, a member of the unikonts, in which matrix proteins contain a PTS1 and are targeted by Pex5 into peroxisomes [49]. This might also be the case in the red alga *Cyanidioschyzon merolae*, in which no PTS2-like sequences have been identified *in silico* [50]. A genomic screen of fully sequenced genomes and available database entries of uncompleted genomes has resulted in the observation that the PTS2 import pathway is not only absent in *P. tricornutum* but also in diatoms as a whole. This is most obvious in the case of 3-ketoacyl-CoA thiolase, an enzyme involved the degradative pathway of fatty acid beta-oxidation. In all representative organisms (except *Naegleria gruberi*) in which the PTS2 import receptor Pex7 is present, the totality of peroxisomal thiolases harbor PTS2 as a targeting signal, whereas in organisms lacking Pex7, these enzymes are targeted by PTS1 into peroxisomes (table 3). Thus, this enzyme is exemplary for a targeting switching from PTS2 to PTS1. Furthermore, orthologs of the PTS2 receptor protein Pex7 could not be identified in other diatoms (table 3). In summary, these data may indicate that both mechanisms already existed in a common eukaryotic progenitor and were lost during the subsequent course of speciation into separate evolutionary groups.

### Conclusions

Is there a reason for the loss of PTS2 targeting sequences and the targeting switch to PTS1? In general, a precise intracellular targeting of proteins is crucial for their biological activity. This is guaranteed by specific targeting signals, which are recognized by receptor-like proteins. Once established, the evolution of a novel targeting signal type might be exceedingly rare. As for peroxisomal proteins of most organisms studied, PTS1- and PTS2-dependent pathways for import into the matrix must necessarily exist in parallel. Based on this data, it is justified to speculate that the progenitors of modern, peroxisome-harboring eukaryotes already used both pathways, so the situation in diatoms described here may indicate a secondary loss of the PTS2-dependent import of proteins into the matrix. Acquisition of a PTS1 seems to be uncomplicated, because it is a variable and short-length sequence at the C-terminal extremity of the peroxisomal proteins. Although *de novo* formation of the sequence may not be too complicated, the factors involved in the selecting against PTS2 in diatoms is enigmatic. The reason for that might not be a disturbance in the functionality of proteins by a PTS2 extension, as several proteins are present in diatoms, which are transported into the matrix via PTS2 in nearly all other known examples. Thus, targeting per se might be the clue. One of the dominant differences in cell morphology between the five major groups of Plantae, Unikonta, Excavata, Rhizaria and Chromalveolata is the presence and the type of plastids. Diatoms, belonging to the group of chromalveolates, harbor complex plastids surrounded by four membranes [23], and consequently nucleus-encoded plastid proteins possess bipartite organized N-terminal targeting sequences [24], [25]. However, the prediction that N-terminally located PTS2 and bipartite plastid targeting sequences could have an influence on one another is not consistent in all cases, because some groups of the chromalveolates with complex plastids, e.g. the brown alga *Ectocarpus siliculosus,* express PTS2-dependent proteins (table 3).

Another indication of the evolutionary loss of PTS2 in chromalveolates can be seen on the level of morphology: the cell wall of diatoms is an extremely complex organized structure, which differs from strain to strain and requires any number of (albeit as yet superficially investigated) concerted targeting events [51]. It is inevitable that such intracellular trafficking and subsequent signaling involved might have been of consequence in the loss of PTS2 at least in diatoms during the course of a signaling switch to PTS1. However, to fully understand this switch, further experimental data are needed, in order to shed light on other factors that may have had an influence on the loss of the PTS2 in diatoms.

## Materials and Methods

### *In silico* analysis and databases

Screening the genome of *P. tricornutum* (http://genome.jgi-psf.org/Phatr2/Phatr2.home.html) for orthologs of peroxisomal proteins was done using known protein sequences from the model plant *Arabidopsis thaliana* and the yeast *Saccharomyces cerevisiae* for BLAST searches [52] using default settings. Predicted gene models from homolog putative proteins were confirmed by either analyzing EST-data or in case of no EST support by RT-PCR (see supporting information S1).

DNA and protein sequences were obtained from different databases: PhatrDBv2.0, http://genome.jgi-psf.org/Phatr2/Phatr2.home.html (*Phaeodactylum tricornutum*), http://genome.jgi-psf.org/Thaps3/Thaps3.home.html (*Thalassiosira pseudonana*), http://genome.jgi-psf.org/Fracy1/Fracy1.home.html (Fragilariopsis cylindrus), http://bioinformatics.psb.ugent.be/webtools/bogas/overview/Ectsi (*Ectocarpus siliculosus*), http://ciliate.org/index.php/home/welcome (*Tetrahymena thermophila*), http://merolae.biol.s.u-tokyo.ac.jp/(Cyanidischyzon *merolae*), http://www.chlamy.org/(Chlamydomonas *rheinhardtii*), http://genome.jgi-psf.org/Volca1/Volca1.home.html (*Volvox carteri*), http://www.cosmoss.org/(Physcomitrella *patens*), http://genome.jgi-psf.org/Naegr1/Naegr1.home.html (*Naegleria gruberi*), http://genome.jgi-psf.org/Triad1/Triad1.home.html (*Trichoplax adhaerens*), http://dictybase.org/(Dictyostelium *discoideum*), http://www.yeastgenome.org/(Saccharomyces *cerevisiae*), http://www.broadinstitute.org/annotation/genome/ustilago_maydis/Home.html (*Ustilago maydis*), http://www.wormbase.org/(Caenorhabditis *elegans*). All other sequences were obtained from the National Center for Biotechnology Information (NCBI) Database (http://www.ncbi.nlm.nih.gov/gorf/gorf.html). Protein domain prediction was done using SMART prediction tool [33]. Protein localization was predicted using the services offered by the CBS-prediction server using default settings (TargetP v1.1; http://www.cbs.dtu.dk/services/TargetP/). Prediction of putative peroxisomal targeting signals was done using the PTS1 predicton tools http://www.peroxisomedb.org/diy_PTS1.html and http://mendel.imp.ac.at/mendeljsp/sat/pts1/PTS1predictor.jsp and the PTS2 prediction tool http://www.peroxisomedb.org/diy_PTS2.html. Putative peroxisomal protein sequences were also inspected manually as the prediction programs provide sometimes only low support for non-canonical PTS sequences.

### Gene amplification and confirmation of predicted gene models

Isolation of genomic DNA and total RNA was done using standard procedures [53]. cDNA synthesis was carried out using 1 µg total RNA using Superscript II Reverse Transcriptase (Invitrogen, Karlsruhe, Germany) according to the manufacturers' instructions. Amplification of selected gene sequences was done using standard polymerase chain reactions using either genomic DNA or cDNA from *P. tricornutum* and *A. thaliana* as templates (for oligonucleotides see table S1). Oligonucleotides were obtained from Sigma-Aldrich (Steinheim, Germany) introducing 5′ and 3′ specific restriction sites. In case of no EST-support predicted gene models were confirmed by cDNA analysis. Amplification of 5′ and 3′ ends was done using standard polymerase chain reactions using *P. tricornutum* cDNA as template. Amplification products were cloned into pJET (MBI Fermentas, St. Leon-Roth, Germany) and verified by sequencing (for oligonucleotides see table S2).

### Cloning and *in vivo* localization of GFP fusion proteins

For *in vivo* localization studies *gfp* was fused downstream of *pex*3 and *pex*10 and upstream of PTS1 containing genes using specific restriction sites. Deletions and additions of PTS1 and PTS2 sequences were done using specific degenerated oligonucleotides (see table S1). Sequences were cloned full length with *gfp* into pPha-NR vector (a derivate of pPhaT1 [54], Genbank accession no. JN180663). The pPha-NR vector contains one multiple cloning site under the control of an endogenous nitrate reductase promoter, which can be regulated by a switch from ammonium- to nitrate-containing medium. Fidelity of amplification and cloning was verified by sequencing all constructs.

Transfection of *P. tricornutum* was performed as described previously [55]. Expression of the fusion proteins was induced 24 hours prior analysis by switching the nitrogen source from 1.5 mM NH_4_^+^ to 0.9 mM NO_3_^−^. Localization studies were performed with a confocal laserscanning microscope Leica TCS SP2 at room temperature in f/2 culture medium using the HCX PL APO 40x/1.25−0.75 Oil CS or PL APO 63x/1.32−0.60, Oil Ph3 CS objectives, respectively. GFP and chlorophyll fluorescence was excited at 488 nm. The fluorescence was filtered by a beam splitter TD 488/543/633 and detected by two different photomultiplier tubes, with a bandwidth of 500–520 and 625–720 nm for GFP and chlorophyll fluorescence, respectively.

### Immunolocalization studies

*P. tricornutum* cells expressing either Pex10-GFP, GFP-trans-2-enoyl-CoA reductase or GFP-3-keto-acyl-CoA thiolase fusion proteins were harvested via centrifugation at 1,500× g and cryo-immobilized by high-pressure freezing on gold carriers (EMPact 2, Leica Microsystems GmbH, Wetzlar, Germany). Subsequently, the cells were freeze-substituted with acetone in combination with 2% OsO_4_, 0.25% uranyl acetate and 5% H_2_O (A.O.U.H.) [56], [57], [58], [59]. Freeze substitution was carried out in the automated AFS2 unit (Leica Microsystems GmbH, Wetzlar, Germany) at −90°C for 4 h, −60°C for 8 h, −30°C for 8 h and then held at 0°C for at least 3 h. The heating time between each step was 1 h. After washing the samples in ice-cold acetone, they were gradually infiltrated in Epon at room temperature, followed by polymerization at 60°C for three days. Ultrathin sections of embedded samples were collected on uncoated nickel grids (400 square mesh). For immunolocalizations ultrathin sections were labeled with primary antibodies against GFP (goat-α-GFP, Rockland, Gilbertsville, USA). As secondary antibodies, rabbit-α-goat IgG coupled to 20 nm gold were used. The procedure for immunolabeling on ultrathin sections was described previously [58]. Transmission electron micrographs were either taken on a JEOL 2100 TEM operated at 80 kV in combination with a fast-scan 2K ×2K CCD camera F214 (TVIPS, Gauting, Germany) or on a Zeiss CEM 902 operated at 80 kV equipped with a wide-angle Dual Speed 2K CCD camera (TRS, Moorenweis, Germany).

## Supporting Information

Figure S1 **Ultrathin sections of** ***P. tricornutum*** **in Epon without antibody labeling.** *P. tricornutum* cells expressing either GFP-trans-2-enoyl-CoA reductase (A, B) or Pex10-GFP fusion proteins (C, D). The boxed areas in (A) and (C) are shown at higher magnification in (B) and (D). CW, cell wall; G, golgi apparatus; Mt, mitochondrium; P, peroxisome; Pl, plastid. Scalebars represent 1 µm.
(TIF)

Figure S2 **Immunolocalization of peroxisomal GFP-fusion proteins in** ***P. tricornutum*** **.** Immunolabeling of GFP-3-keto-acyl-CoA thiolase. The boxed areas in (A), (C), and (E) are shown at higher magnification in (B), (D) and (F). The 20nm gold particles, coupled to secondary antibodies are visible within the peroxisomal compartments (arrow heads). Primary antibodies were diluted 1∶500 (A-D) and 1∶1000 (E-F). CW, cell wall; Mt, mitochondrium; Nu, nucleus; P, peroxisome; Pl, plastid; arrow head, 20 nm gold. Scalebars represent 1 µm (A, C, E), 500 nm (B, D) and 200 nm (F).
(TIF)

Table S1 **Oligonucleotides used for amplification of genetic constructs.**
(DOC)

Table S2 **Oligonucleotides used for amplification of cDNA ends.**
(DOC)

Supporting Information S1 **Full length peroxisomal proteins from** ***P. tricornutum*** **, their putative targeting signals and EST data.**
(DOC)
