# Supplementary figures and images for: A Single Peroxisomal Targeting Signal Mediates Matrix Protein Import in Diatoms
Source: PLoS One. 2011 Sep 22;6(9):e25316. doi: 10.1371/journal.pone.0025316 (PMC3178647; doi:10.1371/journal.pone.0025316)

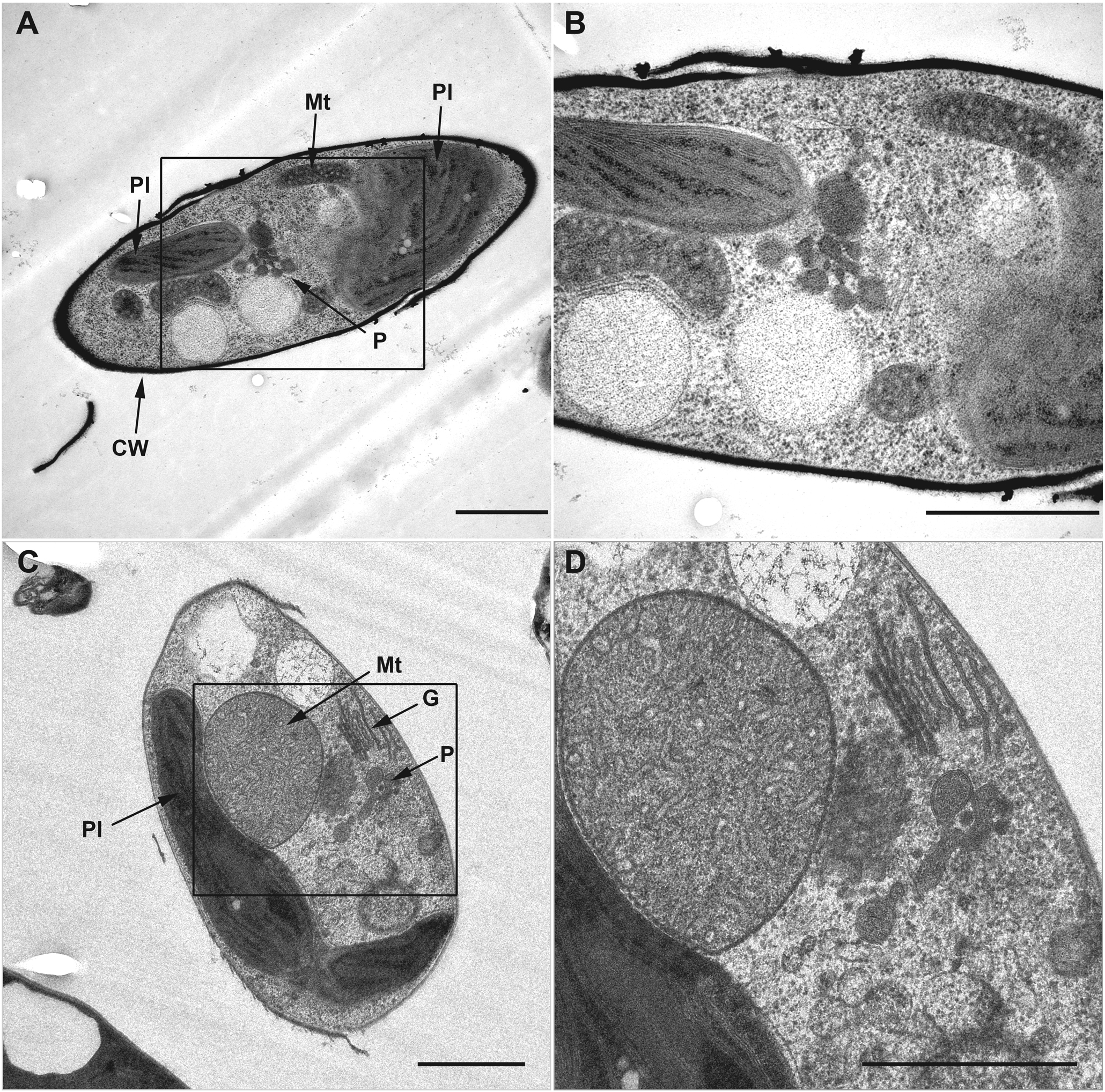

Supplement: Figure S1 — Ultrathin sections of P. tricornutum in Epon without antibody labeling. P. tricornutum cells expressing either GFP-trans-2-enoyl-CoA reductase (A, B) or Pex10-GFP fusion proteins (C, D). The boxed areas in (A) and (C) are shown at higher magnification in (B) and (D). CW, cell wall; G, golgi apparatus; Mt, mitochondrium; P, peroxisome; Pl, plastid. Scalebars represent 1 µm. (TIF) [file pone.0025316.s001.tif]

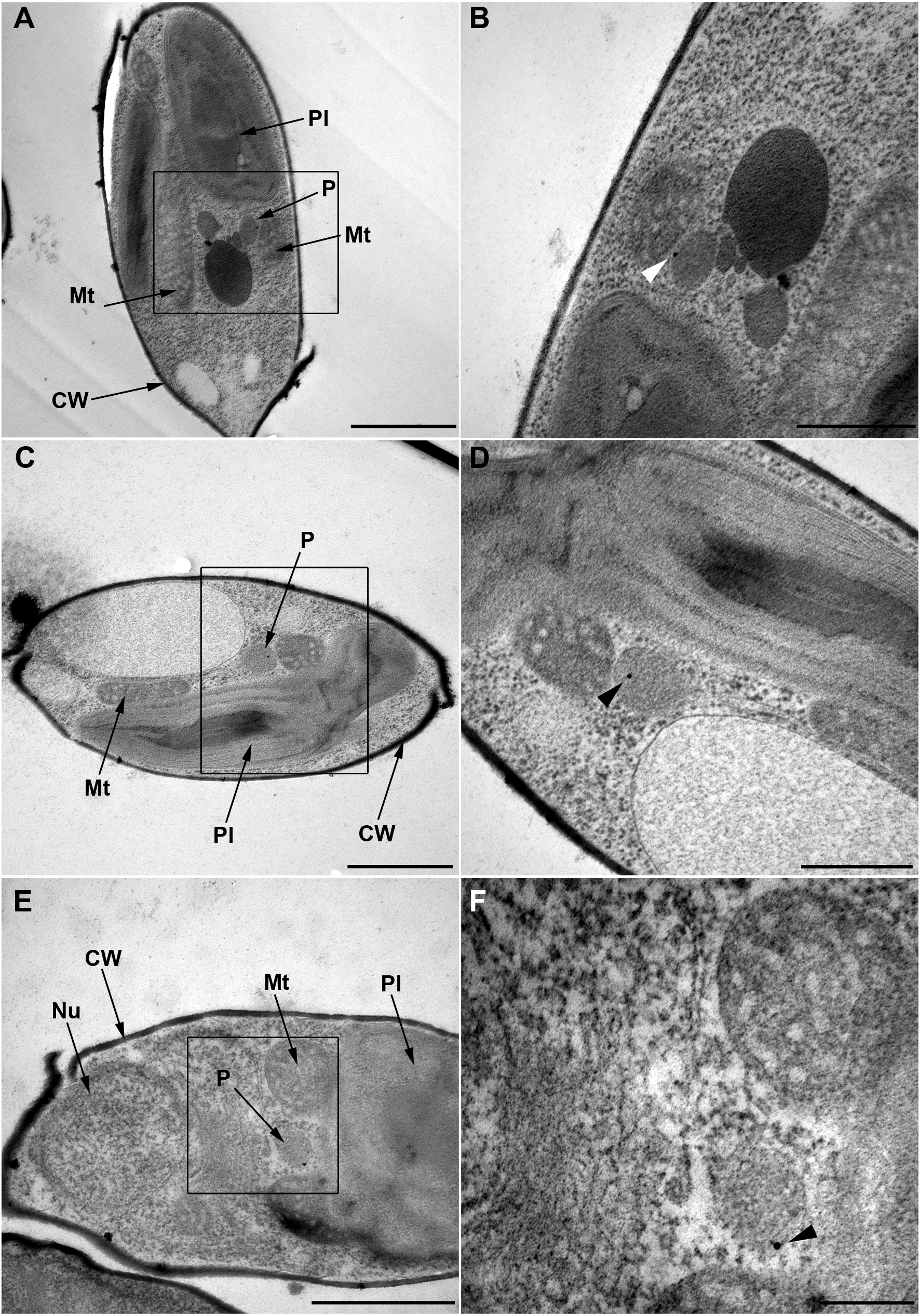

Supplement: Figure S2 — Immunolocalization of peroxisomal GFP-fusion proteins in P. tricornutum . Immunolabeling of GFP-3-keto-acyl-CoA thiolase. The boxed areas in (A), (C), and (E) are shown at higher magnification in (B), (D) and (F). The 20nm gold particles, coupled to secondary antibodies are visible within the peroxisomal compartments (arrow heads). Primary antibodies were diluted 1∶500 (A-D) and 1∶1000 (E-F). CW, cell wall; Mt, mitochondrium; Nu, nucleus; P, peroxisome; Pl, plastid; arrow head, 20 nm gold. Scalebars represent 1 µm (A, C, E), 500 nm (B, D) and 200 nm (F). (TIF) [file pone.0025316.s002.tif]
