# Supplementary material for: A Single Peroxisomal Targeting Signal Mediates Matrix Protein Import in Diatoms
Source: PLoS One. 2011 Sep 22;6(9):e25316. doi: 10.1371/journal.pone.0025316 (PMC3178647; doi:10.1371/journal.pone.0025316)
Supplement: Table S1 — Oligonucleotides used for amplification of genetic constructs. (DOC) [file pone.0025316.s003.doc]

| *P. tricornutum* | long chain acyl-CoA ligase_BamHI_fw | GGATCCATGACGAACCAAAATATG |
| --- | --- | --- |
|  | long chain acyl-CoA ligase_XbaI_rv | CTACCACAATAGTAATCTAGATCAGG |
|  | trans-2-enoyl-CoA reductase_BamHI_fw | GGATCCATGTCCACACCTTGGAAG |
|  | trans-2-enoyl-CoA reductase_XbaI_rv | GTTAATCGATCTAGATTTGTAAAGTG |
|  | malate synthase_SacI_fw | GAGCTCATGATTGAATTTCGTTCG |
|  | malate synthase_XbaI_rv | TCTAGACTACAGCTTGGCGATTCG |
|  | 3-keto acyl-CoA thiolase_BamHI_fw | GGATCCATGAAGAAAGATGACGAC |
|  | 3-keto acyl-CoA thiolase_HindIII_rv | AAGCTTACAGACTAGAACTTGGTT |
|  | 3-keto acyl-CoA thiolase_ΔSSL_HindIII_rv | AAGCTTTTAACTTGGTTCAGCTTCAATTATGG |
|  | Peroxin3_EcoRI_fw | GAATTCATGAGCTCAAAAACCAAAATGC |
|  | Peroxin3_BamHI_rv | GGATCCACGAAAAAACTGTTGGACGTCG |
|  | Peroxin10_EcoRI_fw | GAATTCATGGCTGAATCTGAGGATTTGA |
|  | Peroxin10_BamHI_rv | GGATCCGCGCTTGACGCTGTACTCGCCG |
|  |  |  |
| *A.thaliana* | 3-keto acyl-CoA thiolase_EcoRI_fw | GAATTCATGGAGAAAGCGATCGAG |
|  | 3-keto acyl-CoA thiolase_BamHI_rv | GGATCCGCGAGCGTCCTTGGAC |
|  | 3-keto acyl-CoA thiolase_BamHI_fw | GGATCCATGGAGAAAGCGATCGAG |
|  | 3-keto acyl-CoA thiolase_SKL_XbaI_rv | TCTAGACTAGAGCTTGGAGCGAGCGTCCTTGGAC |
|  |  |  |
| GFP | GFP_EcoRI_fw | GAATTCATGGTGAGCAAGGGCGAG |
|  | GFP_PTS1_HindIII_rv | AAGCTTTTAGAGCTTGGAGTGGGGGGCTTGTACAGCTCGTCCATGCCG |
|  | GFP_PTS2_EcoRI_fw | GAATTCATGCGTCTCCAGGTCGTCCTCGGACACCTCATGGTGAGCAAGGGCGAGGAGC |
|  | GFP_SacI_rv | GAGCTCCTTGTACAGCTCGTCC |
