# Supplementary material for: A Single Peroxisomal Targeting Signal Mediates Matrix Protein Import in Diatoms
Source: PLoS One. 2011 Sep 22;6(9):e25316. doi: 10.1371/journal.pone.0025316 (PMC3178647; doi:10.1371/journal.pone.0025316)
Supplement: Table S2 — Oligonucleotides used for amplification of cDNA ends. (DOC) [file pone.0025316.s004.doc]

| *P. tricornutum* | acyl-CoA oxidase_3'UTR_fw | GGATTGCATTCGTACTGATGCGG |
| --- | --- | --- |
|  | acyl-CoA oxidase_3'UTR_rv | GAAAGACATCCGCATCTCAACAAGGC |
|  | acyl-CoA hydrogenase_5'UTR_fw | GGTCCTGCTTTTCTCGGTAAGCTTCC |
|  | acyl-CoA hydrogenase_5'UTR_rv | GCTCGATTCTTAAGCCGATTTATGC |
|  | acyl-CoA hydrogenase_3'UTR_fw | CCATTGTAGCGGTGCCCTTGCC |
|  | acyl-CoA hydrogenase_3'UTR_rv | GCATCTATCGATGAAGGTTGCTCACCG |
|  | trans-2-enoyl-CoA reductase_3'UTR_fw | GGGAATTATTTGGACGGAGAGCGG |
|  | trans-2-enoyl-CoA reductase_3'UTR_rv | CCACACAGTTGAGCATGTGCTACG |
|  | 3-keto acyl-CoA thiolase _3'UTR_fw | GATGCCGATTCAGCAACCTTGGC |
|  | 3-keto acyl-CoA thiolase _3'UTR_rv | GTCAAGTAGAGTACCGGACGGGAGATCG |
|  | long chain acyl-CoA ligase int_EcoRI_fw | CGTACCAGAATTCTTTGGTTGGC |
|  | long chain acyl-CoA ligase_XbaI_rv | CTACCACAATAGTAATCTAGATCAGG |
