# Supplementary material for: A Single Peroxisomal Targeting Signal Mediates Matrix Protein Import in Diatoms
Source: PLoS One. 2011 Sep 22;6(9):e25316. doi: 10.1371/journal.pone.0025316 (PMC3178647; doi:10.1371/journal.pone.0025316)
Supplement: Supporting Information S1 — Full length peroxisomal proteins from P. tricornutum , their putative targeting signals and EST data. (DOC) [file pone.0025316.s005.doc]

>, name; [] protein ID (http://genome.jgi-psf.org/Phatr2/Phatr2.home.html); description of gene model EST-support; **bold fonts**, putative peroxisomal targeting signal 1; underlined fonts, SignalP signal peptide prediction by NN (neural networks); italic fonts, SignalP signal peptide prediction by HMM (hidden Markov models)

>catalase, [22418], gene model fully supported by ESTs

MSDQTSSRDAASNQLDAYASQRYVVSPAIELLKASNGAPVDSLTASMTAGPRGPIVLQDFTLLDHMARFDRERIPERVVHAKGAGAFGVFQVTSPEPIRSVCKAQVFQSMEPTPVAVRFSTVGGETGSADTARDPRGFAVKFYTTEGNWDLVGNNTPIFFIRDPMLFPSFIHTQKRNPATHLADPDAFWDFIGLRPETTHQVTFLFSDRGTPDGYRHMNGYGSHTFKNVNSNGEAVYVKYHWKTDQGVANLDAEHAARLAASDPDYATRDLYNAIATGNYPSWTLYVQIMTYAEAAAVDINPFDLTKVWPHSRFPLHEVGRMTLNRNPKDYFAEVEQLAFAPSHLVPGIEPSPDKMLQARLFSYPDTHRHRLGTNYQAIPVNAPRNISAAQQHNYQRDGPMQVTENGAGAPNYFPNSFGGVQPPPATAHGPELAWHADTVSGQVVRIDTGDEDNVSQCRDFYRNVLDQAARARLTDNLAAHLCRAQPFLRARAIHNFTQVDSTFGAQLKAAVAQKVMHTPPPSQSRRRPAKLNPPRIVPPPTASRGLCPAGYGNQGSPAHGYA**SKL***

>bifunctional enzyme, [55069], gene model supported by ESTs

MTRSQVVSSSSQGLPTDTTAVICLADAAAGASPLHPLNAKLRLYLLQQLRAAEDNPAVTSLILTGGGPNFSAGADLTEFASLIPAAATTVPTPSDVGHHHPHAAPSLIDVVTAIEACRKPIVAAIDGVCLGGGCELALACHARVATARARLGLPEVHVGVIPGAGGTQRLPKLVGLRQALPMILQGSTVSAASALRMGLLDAIAPEATADSLRTTARQWAAYGEVLPTIRRTGDLQRPESPAEAHALLHVAELSLPPLGSHGMRAAIQALRASGTPIQHGMRRSPQGRARRHVFFATRRAQKVWSPVSNAVPTAGPPHGLWSKDDAQSAVPVAVVGAGTMGSGIALVLLQAGFHVTLVDVHAPALAKGMEFLKRTLASMVQRRQLKPTQLTALEAKLRATSNLQELSQCLLVVEAVVEKLIVKQSIFATLDKVTPPTALLLSNTSTLDIDAMASAVSSRRRGLFAGWHFFSPAHRMKLVEIVRGSATSDDTTALLQALTKQIGKIGVVVGNCDGFCGNRMLKPYSAETVLLLTETQTTVAAQDYAIRGVYGMALGPFEMADLAGNDVGYNIRVERQWARRARDDPLPPNRPARYTELPDVMISDYGRLGQKVGKGWYDYDSNIGKGRKPLPSSEMDALIRRYLAPKPSPALVAAEIIERVLYPLINEGFKCLEESIVRQPSDIDVVYVYGYGWPVWRGGPMYAADHEIGLPRLLRTLRELSKQFPTTEHYVPSALLVECVARKVTVEEYYQKNYHTASTGSAML**SKL***

>long chain acyl-CoA ligase, [17720], gene model not fully supported by ESTs but confirmed by cDNA

MTNQNMRSTVLPQTDRHDRGEIRRSWNPVVNAELGVYGCRTMYEGFRRGAKVNPLGPCMGFRAVSTTGFATPFIYSSYTECLARIDAFAAGLDTLKLIQPNEDGLAVLALYMKNCMEGAIGEHGAFAIGGSSVLMYDTLGPDTVTFILDQTSARSVVCTRAELPKLCESKVSGKCPAFTAAILVDGVTEDAAKMALEAGLEVLSFAKVEAVGAVCIAEKGHHHRPPAPTDVAAFCYTSGTTGNPKGALLTHENIMSAIAGFYGAVGDLEAQPFDRHLSYLPLAHIFERIVSSQMFCAGASVAFFRGDPTLLIEDMQACRPTVMPVAPRVLNKIYDKIQAGISSVGGLKKKLFDAAVAAKAEGLQSGHMKHALYDRLIFNKIKKGLGMDQLRMMVSGSAPLNDTVMTFFRCMLAIPVVEGYGQTEGAAAATIGSSDDMATVGHVGGPVGSVEIVLVDVPEMGYFHTDTLHRGMACQGRGEICVRGPSVFKGYYKDDLNTRETIDNEGWLHSGDVGLWRPDGNLQIIDRKKNIFKLSQGEYVAPEKIENFLISSPLIGQCFVYGDSYQNSLVGLMVPDEEPVRTWAAVNAPELKSASLAEMCNSEALKAAVMADIQRIGVESRLLGFEKPKDILLVSVPFTIENEMMTPTFKLKRQKIRDTYEKEIDLLYAGLPPPL**SKL***

>carnitine-o-acetyltransferase, [48078], gene model fully supported by ESTs

MWRRGRMSSITSTVTTMATTTRRRANARRILSTSVPLRPWKTSVVESHGDYRAEAWLEDHVGGDLYRYQADLPMLPVPTLAHTLERLLPTVLPVCRHDEEMESLRQAVDRFPEQASTLQERLLHRQQQHENANSSWLQHWWNTLGYLQVRESVVINVSYFFHLADDPSATTLTQRGAALLTAAAQYRHQVCSGSLAPTVLGRGERAQPLCSAAYKYMFHASRIPRPQQDSYKIYDPARYRHAVVARKGHFYTLNLCGPDHATPYPVATLQAGLERIVAHADAMHAHPPPPQLGWLTTAPRDDWAAARQTLLDTDQQYQRSTVSDALERLESGAVLLCLDDVHAVSRLEMGQLLLHGAGHNRWFDKSVQLVVTENGKAGLIGEHSMMDGMPMVGLADHCTKVTYEQCLRKSPTGTMVPEPTVEPIFNTPEFTQILQDPIVHNLVDQGTCCVAKMDCDDWTGRHAMQSQSFQGYGSQFIKQAGFSPDAFVQMAMQLATYRLWGEQAGTYEATQVRPFRHGRTETTRTVSLESAAFVQRLGLRPQYNEHDAEVRGEKLRLLRDAVQAHVRYIGAAAQAQGVDRHFFGLSMLVADGEKAPDLYAHPAFVRAKRWRVSTSHLTHPKIVNWGYGEVVPDGVGLSYSIHPRHCVFNVTALKETGWAEKLCTLLEESLLELRTLIEMDQAPPS**SKL***

>glycolate oxidase, [22568], gene model fully supported by ESTs

MLEESEKRNLLNVDDYQVLAKTKLPHSLYEYLASGTADATTLRENRDAFARWYLRPRAMRPVGRISTRMVLFGQGLSMPVFCSPAGVHALCHPDGECATARVCQDLGLLFGLSQHATKSIEQVAAAAPQSHRYYQAYILKDRSITARLVQRAIQAGYSGIFLTVDSVRFGYREADARNGFDALPSPHRLANYDEVRQQNLDQTYNAKTHLAWDQNSELLFEQNVSWKDVTWLKEEVCGGLPLIVKGIMTAEDAVLAIEAGADAIMVSNHGGRQLDTCLGSIDVLPEVVMAVGGRVPVLLDGGVRRGTDVVKALALGAAAVGLGKPLFFALACGGESSLKDMLEILQTEIEVAMALCGCETISDIQSSHITRHPGGHFQ**SRL***

>acyl-CoA oxidase, [19979], gene model partial supported by ESTs, incomplete at the 3`end

MPVNEDLRRERARATFDVKQLTHMLDGGSDRTARRRQLQAIIERDPTGIFSNDDNAYLHRTDRHVRGLAKHVRMVELCRTLGIGDKYDGQVVMDPDFPVLLAALADDLPTALHWIMFVPNIVSLCDEEQQRAWLPLCRDWKMIGCYAQTELGHGSNVRALETTATFVSEDQDGTKGGSFVINSPTITSAKFWPGTLGRTANHAMVIARLIDGHGKDQGMHNFLVPLRSMKDHKLLPGVKSGDIGPKIGYNVMDNGFAQFDNVKIPRRNMAMRFAHVDERGRYSKKSVSAAASKISYITMMQVRAHICNEAGKNLAVACTIVTRYSALRRQGYASDGESELQILDYKQQQHRVFPLIAASYCFFFTGKRVMEQLKDIEDKLVSNKSISKAAVTDIHASSSALKSFTTTVAADGIEDCRKACGGHGFLQCSGLPELITTYLQSPTVEGDNQMLPQQVVKVLLKLVQTVQDDGDLSDYVSCDSYGLISSLQSNLKGVKEDCRIVSEIDVMNLDALLAALCHRSARLLVGVASQIQGSISSGKSYQEAWNDALVEMARVSRAYSQFLLLRNFMEGIDGEERSGAIGVAEVTVLQNLARLFALYWMEKELGDFLEDGYVTADHSRWVRSAVLQLLDCIRTDAVSLVDARDFSDFKLKSALGRYDGDVYPAILKAALRDPLNHSDPGPGYEQHLKRLIAGGTGTYKATV**SRL***

>acyl-CoA dehydrogenase, [42907], gene model partial supported by ESTs, incomplete at the 5`end

MNSASLTYLRPDVAALKKRMDDFVELECIPAEAEYEAHMKDRNGADRWTMEAVPPCINRLKNRARELGLWNLFVPPHLISRIPERALAPAVALSYREYGILCESLGRAPTVAPEACNTSAPDTGNMEVFLEFGTPAQKTTYLIPLLQGQIRSAFLMTEPDVASSDPTNLETKLTKKISNGTVEYILTGRKWWSTGAMDPRCRVALVVAKMDYSDPSCQAQPQTSKHGAHTIVAVPLPHPQVIMQRPLTVFGYDDAPHGHAEVVLNGVRLDESDLIFGEGSGFRVSQARLGPGRIHHCMRALGIATRSYELMLQRTMERKTFGKYLWEHGGCQDAIADSASDLEAARLLTLSCAAAMDDVGVKNARDKIGIIKVTVPELTYRVVDRAIQVFGGAGVHEDLFLARALAGLRTLRIADGPDAVHRRTVALMEIKKRALRGSQH**SRL***

>trans-2-enoyl-CoA reductase, [37372], gene model partial supported by ESTs, incomplete on the 3' end but confirmed by cDNA

MSTPWKSCFRDGLLEGKVALVTGGGTGIGLSIATELASLGAIVVIASRNRQTCQEAADRMNATQVSGKIVAGPSTSIRKEDEVRNLIAWVLESFDALHLLVNNAGGQFISAAEDISKGGFSAVVETNLTGTFLVCREAFTQYMDKHGGAIVNITLGNRNGMPMMSHSGASRAGVENLTATLSTEWMESNVRVNCVRPGIIWTESGFENYGPAGEMFVERLLPAMPARRFGSPEEVSSAVVWLLSEGASYVTGTVLNVDGASAYTLLPLRDIEDKEHLPFY

GTLPRK**ARL***

>malate synthase, [54478], gene model fully supported by ESTs

MIEFRSEQVHVRVHAPANKAAEEMLTPDALRLLGLLCERFDVRRQALLAARKTHATSFDAGDVPHFLSAEDHPAQRDPHWRCAPVPDDVQDRRVEITGPVDRKMVINGLNSGACVYMADFEDSTSPTWFNVIDGQLNLRDAVRGTIAFTNAAGKVYTVQHASRPATLFVRPRGWHLDEAHVTVNGKVASGSLFDFAMYFFHNVHHLKEKGTGPYFYLPKLESHKEAALWNDVFVAAQQFMGVPIGTIRATVLLETITAAFEMEEILYELRDHSLGLNCGRWDYLFSFIKKFKHHTDKLTPDRNHLTMTTPLMEAYVKRLIYICHKRGTFAMGGMSASIPIKNDPAANDAAMQKVADDKLREVTAGHDGSWVAHPALVKVAKDVFDEHMLTPNQITSKPGYVGSSINEQDLLRLPPIPHGKAITSEGLARGVGIVLAYTEAWLRGIGCIPLHNAMEDAATAEISRAQIWQWRSQKASTQDDNRPITASRVAALVQQEVDRQCNGVAGKSKGKWRLAGNLVENMLNKDELDDFLTSVCYPHIVTTAYDDGRI**AKL***

>3-ketoacyl-CoA thiolase, [41969], gene model partial supported by ESTs, incomplete on the 3' end but confirmed by cDNA

MKKDDDDIVIVSALRTPMCRSRKGGLANVLPSTLFQTVLEATLQATQLPPRDVEDIVVGNVLMPPSGFAALRMAQIISGIPETTSLQTVNRQCASGLQAVANVANSIAANEIQIGIGAGVESMSLYPMNTIKPPQVDWETMQTSRTAMDCLLPMGITSETIVRKYGLKREDLDAFAVTSHKKASAAQRSGKFSAEIVPVGDISQDDGIRPDADSATLARLKPAFSKSGVTTAGNSSQTTDGAAAVVLMKRKEAQRRGLKIMGVWRGYATAGVPPQIMGIGPAVAIPKVLELTGLSKSDIDLFEINEAFASQATWCVDELGLDWDKVNPNGGAIALGHPLGCTGARMVATLLHELHRRKRRYGVISMCIGTGMGAAAIIEAEPS**SSL***

>Pex10, [47516], gene model fully supported by ESTs

MAESEDLIPESLLAIAKDSRYVQECCEVVVEIWHRWCPRQRRENLERDAWFWSLVLYIVLVVGSRGRTLGMEALGLSYINRDASRTSSSFISRFKLLSTSLGLVAAVYLLQSQTSETDATRERVENLTGSSRRDFFEAQRRAMIQRSRQPSSDSSETLVRSQSMPMPASLFLAEWKQKAKQLVQGVAKALLPYNDLTHGPHDLPDQHSQSESPSQTIATWLIRFHLGLYCLNGRYPSWLHRILGHQLHSVDPQSSRLVNKPTSVRIVGLLLLSQSAAAALLGFSRVLLRWWIDTRDGPSLSTNVRGSSIKFIGARHSAAHNPGVVESNTSCAICRQPRRHPACPVTCGHCFCWSCLQSWIMTRGECPLCRVKCTPSQVLALYRYAPATPGATVTSRGEYSVKR*

>Pex3, [50623], gene model fully supported by ESTs

*MSSKTKMRRKKQSISLGSVISAAAVAYG*TYKVADWAWNRYVTKRKKNDYQVNAAIATSFMNFLCSQTSVGAHAEDGVASHIDHIPGPNRRLRMRRQRMTRCRQEAAQALRGFSPALRSIVELHTNTAQATRLLKQLRANRTTEKHATSRRSEEQALWKEIQRKTMTRMLTTAYAHTILFLVLTTQVNLLGGRLFEESLQNTSLSSNVSMSNDSVASDRMVSYQESHRFVLQHTYDYFLNKGVHSLLSTVEQAVDSVLGGWNVFDKACLHISREQFDCALVKIRGLIEGGLRTDVSRTSGRSSRRESILRFLMPSSILEHSIQDDLARSILDETWDLVESPVFSDAQQECLNATFASMRDRFWGKIFDDNGLSGTKPWAHFFR*
